# Fast Isocenter Determination Using 3D Polymer Gel Dosimetry with Kilovoltage Cone-Beam CT Reading and the PolyGeVero-CT Software Package for Linac Quality Assurance in Radiotherapy

**DOI:** 10.3390/ma15196807

**Published:** 2022-09-30

**Authors:** Piotr Maras, Marek Kozicki

**Affiliations:** 1 Department of Radiotherapy Planning, Copernicus Hospital, 93-513 Lodz, Poland; 2 GeVero Co., Tansmana 2/11, 92-548 Lodz, Poland; 3 Department of Mechanical Engineering, Informatics and Chemistry of Polymer Materials, Faculty of Materials Technologies and Textile Design, Lodz University of Technology, 90-543 Lodz, Poland

**Keywords:** isocenter, radiotherapy, polyGeVero-CT, 3D polymer gel dosimetry, quality assurance, QA, computed tomography, iCBCT, CBCT, PABIG^nx^

## Abstract

This work presents an approach to the fast determination of a medical accelerator irradiation isocenter as a quality assurance (QA) procedure in radiotherapy. The isocenter determination tool is the tissue equivalent high-resolution 3D polymer gel dosimeter (PABIG^nx^) in a dedicated container combined with kilovoltage imaging systems and the polyGeVero-CT software package (v. 1.2, GeVero Co., Poland). Two accelerators were employed: Halcyon and TrueBeam (Varian, USA), both equipped with cone beam computed tomography (CBCT) and iterative reconstruction CBCT (iCBCT) algorithms. The scope of this work includes: (i) the examination of factors influencing image quality (reconstruction algorithms and modes), radiation field parameters (dose and multi-leaf collimator (MLC) gaps), fiducial markers, signal averaging for reconstruction algorithms and the scanning time interval between consecutive scans, (ii) the examination of factors influencing the isocenter determination, image processing (signal averaging, background subtraction, image filtering) and (iii) an isocenter determination report using a 2D and 3D approach. An optimized protocol and isocenter determination conditions were found. The time and effort required to determine the isocenter are discussed.

## 1. Introduction

In recent years, two types of advanced linear accelerators have been introduced to radiotherapy, having the following built-in systems: magnetic resonance (MR) and kilovoltage imaging, which is capable of cone-beam computed tomography (CBCT). Due to constant demand for improving image quality and reducing image noise, iterative cone-beam CT (iCBCT) was introduced to accelerators [1,2,3,4,5]. This work reports on CBCT and iCBCT using TrueBeam and Halcyon (Varian, USA) accelerators and on determining the irradiation isocenter that should match a predetermined point in space by the use of a 3D polymer gel dosimetry tool.

The radiation isocenter [6] can be determined by the Winston–Lutz test [7] or star shot measurements [8,9,10] by usingfilms (2D flat dosimeters) as well as Electronic Portal Imaging Devices (EPIDs) [11,12,13,14,15,16], which currently are the broadly used methods.

Apart from the above-mentioned tests, a new approach for isocenter determination has been presented using 3D polymer gel dosimetry [17]. It should be mentioned that 3D radiotherapy dosimetry has been studied since the 1980s. For nearly forty years of research, components of 3D dosimetry have been developed, such as: (i) 3D dosimeters: Fricke-based polymer gels and radiochromic gels and plastics, such as radiofluorogenic gels, polyurethane resin-based radiochromics, silicone-based dosimeters, radiochromic gels based on micellar solutions, gels containing tetrazolium salt or leuco dyes and the Pluronic F-127 physical gel matrix [18,19,20,21,22,23,24,25,26,27,28,29,30,31,32,33,34]; (ii) methods, devices and protocols for 3D scanning: magnetic resonance imaging (MRI), computed tomography (CT), optical computed tomography (OCT), ultrasonography (US); (iii) procedures for the use of 3D dosimeters; (iv) containers for 3D dosimeters; and (v) fast processing of results: codes and commercial software packages, as well as 3D dosimetry, have been tested in multiple radiotherapy applications. Aspects of 3D radiotherapy dosimetry are discussed elsewhere [17] (and are referenced therein).

It was shown by Dorsch et al. [35,36,37] that the PAGAT 3D polymer gel dosimeter coupled with MR reading can be a tool for measuring the radiation isocenter accuracy by means of a star shot. The results obtained are on a par with those for a radiochromic film dosimeter. The MR evaluation of irradiated PAGAT was possible soon after irradiation (despite the post-effect in the irradiated dosimeter) with acquisition times related to magnetic field strength. In turn, the ability to visualize the dose distribution in 3D and isocenter verification with kilovoltage CBCT was shown elsewhere for the NIPAM polymer gel dosimeter and data processing using in-house developed code in Matlab (Mathworks Inc., Natick, MA, USA) [38,39]. The numerous conclusions of this work include: (i) The overall robustness and accuracy of the isocenter verification test performed. Part of the test (imaging and irradiation, apart from data processing and isocenter calculations) can be performed in about 40 min. (ii) Few-day longevity (storage time) of NIPAM related to the storage temperature. This, in our opinion, may be related to the type of container used and its oxygen permeability, as mentioned by the authors, over a longer storage time. Nevertheless, NIPAM does not need to be used for isocenter determination shortly after preparation, which is similar to other 3D polymer gel dosimeters when properly stored in appropriate containers. (iii) The time after irradiation determines the contrast-to-noise ratio for NIPAM due to the ongoing post-effect after irradiation (polymerization and crosslinking reactions). The post-effect is typical for 3D polymer gel dosimeters. (iv) Optimized CBCT acquisition parameters are reported as well as data processing that includes background subtraction, causing an increase in the contrast-to-noise ratio. (v) The potential use of the reported isocenter test, which is linac acceptance and commissioning as well as verification after routine maintenance or repairs performed periodically [38].

In this work, 3D polymer gel dosimetry was used as a tool for determining the irradiation isocenter. The tool consists of a 3D polymer gel dosimeter in a dedicated ~1 L container; kilovoltage imaging systems, which are the CBCT and iCBCT of Halcyon and TrueBeam accelerators; and fast data processing using the polyGeVero-CT software package (v. 1.2) [40]. As a 3D polymer gel dosimeter, PABIG^nx^ [41,42,43] was selected. It is a normoxic version of the PABIG polymer gel dosimeter [32,33,41,44]. So far, the following features of PABIG^nx^ are known: (i) extracted from [41]: stability from 20 h to at least 95 h after irradiation, saturation after absorption of about 40 Gy; dose threshold: below 1 Gy; linear dose range: ~18 Gy; dose sensitivity: ~0.1 Gy^−1^ s^−1^; and (ii) extracted from [43]: dose response similar for multi-vial, cross beam and depth dose calibrations; dose sensitivity: 0.0898, 0.0971 and 0.0975 Gy^−1^ s^−1^, respectively; for brachytherapy, dose sensitivity: 0.0787 Gy^−1^ s^−1^; linear dose range: ~18 Gy; dynamic dose response: ~40 Gy. A recent study [42] confirmed the dose response features of PABIG^nx^, and revealed the following: (i) a linear and dynamic dose response of 0.5 to ~18 Gy and 40 Gy, respectively; (ii) dose sensitivity of 0.071 ± 0.001 Gy^−1^ s^−1^; (iii) integral 3D dose distribution for at least 24 days after irradiation; (iv) batch-to-batch reproducibility; (v) dose response, the same for irradiation with 6 MV photons, 15 MV photons and 6 MV photons FFF of 0.0168–0.1094 Gy/s dose rates; and (vi) soft tissue equivalence (it should be noted that the parameters of PABIG^nx^ may differ from study to study due to, e.g., different readout). It was therefore assumed that the features of the PABIG^nx^ dosimeter make it useful for isocenter determination.

As a part of the tool selected in this study, in addition to PABIG^nx^, the polyGeVero-CT software package was used for data processing. It is believed that the calculation complexity of 3D radiotherapy dosimetry data should be reduced to using ready, dedicated software instead of raw codes to be written for a particular experiment. This should save time and facilitate calculations. A description of the software and its use in similar studies can be found elsewhere [40]. Version v.1.2 of the software has been enriched with functionalities related to isocenter calculation.

The aim of this study was to develop a procedure for fast isocenter determination using 3D polymer gel dosimeter (PABIG^nx^)-coupled CBCT and iCBCT of Halcyon and TrueBeam accelerators with standard modes, and data processing using the polyGeVero-CT software package. To achieve this goal, it was necessary to investigate: (i) factors influencing CT image quality: reconstruction algorithms (CBCT and iCBCT), CBCT and iCBCT modes, radiation field parameters (field size and dose: monitor units, MU), signal averaging for reconstruction algorithms, fiducial markers and scanning time interval between consecutive scans; (ii) factors influencing the determination of the isocenter: image processing (signal averaging, background subtraction, image filtering) and (iii) isocenter calculation using 2D and 3D approaches on the isocenter parameters. Additionally, the time and effort required to determine the isocenter are discussed.

## 2. Materials and Methods

### 2.1. Preparation of a 3D Polymer Gel Dosimeter

The PABIG^nx^ polymer gel dosimeter was prepared analogously to previous descriptions [41,42,43]. Briefly, poly(ethylene glycol) diacrylate (PEGDA, Mn = 700 g mol^−1^; Sigma-Aldrich, Saint Louis, MO, USA), *N*,*N*’-methylenebisacrylamide (MBA or Bis; Sigma-Aldrich, Saint Louis, MO, USA), gelatine (type A, 300 Bloom; Sigma-Aldrich, Saint Louis, MO, USA), copper sulfate pentahydrate (CuSO_4_·5H_2_O; Chempur, Piekary Slaskie, Poland), ascorbic acid (AsAc; Chempur, Piekary Slaskie, Poland) and double-distilled water were used. For instance, the synthesis of 2200 mL PABIG^nx^ was as follows. A 3000 mL thermal shock-resistant beaker (3 mm-thick glass walls) was used. About 1.7 L of double-distilled water was added to the beaker, and the beaker was placed on a magnetic stirrer with a temperature control unit (IKA, C-MAG HS7, Staufen, Germany). The temperature was set to 50 °C. Then, MBA (88 g, 4% *w*/*v*) was added to the water (at about 25 °C) under steering (2000 rpm). It took no longer than 10 min to dissolve MBA at 50 °C, converting the white dispersion of MBA powder into a clear and transparent solution. Then, gelatine (110 g, 5% *w*/*v*) was added portionwise with vigorous stirring (2000–3000 rpm), and the mixture was stirred until it was transparent. The temperature of the solution was increased to 55 °C, which was necessary to dissolve the gelatine (note that the temperature should not exceed 60 °C, as this may be detrimental for the strength of the gelatine physical gel). It took about 30 min to dissolve gelatine at 55 °C, which was a clear, transparent but light-yellow solution. Next, the solution was cooled to about 30 °C (under continuous stirring). Note that this volume of solution (>1.7 L) may take several hours to cool if the room temperature is around 23 °C. The cooling process can be accelerated by immersing the beaker with the solution in cold tap water or by placing it occasionally in the refrigerator. However, during such cooling, care should be taken to prevent inducing local gelation of the solution near the walls of the beaker. Continuous mixing is recommended. It is not advised to extend the refrigeration of the solution, as this may induce crystallization and/or precipitation of MBA in addition to the gelling of the incompletely prepared solution. Next, 88 g (4% *w*/*v*) of PEGDA was added, which is 78.57 mL, since the density of PEGDA at 20 °C is equal to 1.12 g/mL. Note that PEGDA was stored in a refrigerator, according to the manufacturer recommendations, at 2–8 °C; at this temperature, it is solid (melting point is 12–17 °C). Therefore, it should be taken out of the refrigerator a few hours before the synthesis of PABIG^nx^ to convert it to a liquid and to equilibrate to 20 °C before adding it to the 3000 mL beaker. The solution of MBA, gelatine and PEGDA was then quantitatively transferred to two volumetric flasks of 2000 mL and 200 mL. However, before that, copper sulfate pentahydrate (0.0088 g, 0.0004% *w*/*v*) and ascorbic acid (0.154 g, 0.007% *w*/*v*) were added separately to these volumetric flasks with a small volume of double-distilled water (about 20 mL in each flask) and were mixed manually. The residues in the 3000 mL beaker were transferred to the volumetric flasks by washing the beaker three times with a small volume of double-distilled water (time required for this operation is about 10 min). The remaining volume of both flasks was accounted for with double-distilled water up to the mark of the flask. Afterwards, the solutions in the flasks were poured out to the 3000 mL beaker and thoroughly mixed. After mixing, the solution was homogeneous, and part of it (e.g., 200 mL) was transferred to both volumetric flasks (2000 mL and 200 mL). The flasks were rotated between the hands, and the solution was transferred to the 3000 mL beaker. The described operation was repeated three times (total time required is about 10 min). Eventually, the preparation was completed with a 2200 mL dosimetric solution in the 3000 mL beaker. The preparation and quantitative transfer of the PABIG^nx^ solution carried out in this way allowed for obtaining appropriately dissolved components with fixed concentrations (*w*/*v*). The laboratory procedure of preparing large volumes of a 3D polymer gel dosimeter may be more laborious and time consuming than with small volumes (e.g., 50 or 100 mL), e.g., due to natural reasons such as the cooling rate of large volumes of the dosimeter solution before adding certain components.

The prepared liquid composition of the PABIG^nx^ dosimeter was poured into two types of containers depending on the particular experiments, as described in the sections below: (i) a container of the following dimensions: a height of 210 mm + 10 mm a filler plug, inner diameter of the main part of 90 mm, height of the main part of 130 mm, width max – lower lid stand of 130 mm and volume of approximately 1040 cm^3^ (PH-5-DD1, GeVero Co., Lodz, Poland), and (ii) a container of the following dimensions: a height of 170 mm + 10 mm a filler plug, inner diameter of the main part of 80 mm, height of the main part of 90 mm, width max – lower lid stand of 120 mm and volume of approximately 650 cm^3^ (PH-6-DD2, GeVero Co., Lodz, Poland). The containers were equipped with pressure-compensating valves, which were designed to compensate for pressure changes inside the polymer gel dosimeter during and after filling the containers, as well as during the solidification of the gel dosimeter. Some polymer gel may shrink slightly during the solidification, storage or under the influence of ionizing irradiation. The valve was intended to improve the performance of the dosimeters. Note that improper containers for 3D gel dosimeters can cause gel dosimeter cracking and the formation of voids, which have a negative impact on the CT (or MR) image quality and the calculation of results (e.g., see Figure 7C in [45]).

Note that 2200 mL of the PABIG^nx^ dosimeter solution allowed for the filling of two PH-5-DD1 containers. The containers with the PABIG^nx^ dosimeter solution were kept at 20–23 °C and were protected from daylight for about 20–24 h in order to solidify and obtain full mechanical strength by gelatine. It is not recommended to store PABIG^nx^ in a refrigerator in order to speed up the gelling process. Then, PABIG^nx^ was irradiated and CT scanned. The scanning was intentionally performed shortly after irradiation to reduce the total time of the study, for instance, for isocenter determination. However, it should be noted that the post-effect occurred in 3D polymer gel dosimeters, including PABIG^nx^, which was undergoing polymerization and crosslinking reactions in the dosimeter [41]. These reactions lasted up to about 24 h after irradiation. Thus, the intensity of the CT signal may have been stronger after the end of the post-effect. It could even be much stronger (with respect to the signal after irradiation) already after about 5 h after irradiation, because the dynamic phase of the post-effect was observed in the first few hours after irradiation [41]. Consequently, to boost the measured signal intensity for the irradiated regions of the 3D polymer gel dosimeter, the CT measurements could be postponed after irradiation. The scope of this work, however, did not include measurements at various times after irradiation.

### 2.2. Impact of Reconstruction Algorithms and Modes on Image Noise

The influence of reconstruction algorithms (CBCT and iCBCT) as well as CBCT and iCBCT modes on image quality was investigated for two accelerators: Halcyon (v.3.0, Varian, Palo Alto, CA, USA) and TrueBeam (v.2.7, Varian, Palo Alto, CA, USA). The CBCT and iCBCT acquisition parameters for Halcyon and TrueBeam accelerators are shown in Table 1 and Table 2, respectively. The default modes of CBCT and iCBCT (Table 1 and Table 2) were used as supplied by the manufacturer; no modification of the modes was made.

Image noise (standard deviation, Std, for selected volumes of interest, VOIs: 20 mm diameter, 20 mm length) was examined for PABIG^nx^ in the PH-5-DD1 container (GeVero Co., Lodz, Poland) for the Halcyon and TrueBeam accelerators. The containers were placed horizontally on the accelerators’ couches. The obtained images were processed with the polyGeVero-CT software package (see Section 2.8; GeVero Co., Lodz, Poland).

### 2.3. Impact of Signal Averaging for Reconstruction Algorithms and Selected Modes on Image Quality

The study was performed using the irradiated PABIG^nx^ dosimeter for the TrueBeam accelerator (CBCT and iCBCT, mode Pelvis and Pelvis Large). The dosimeter was prepared as described in Section 2.1 in the GeVero Co., PH-6-DD2 container. Irradiation was performed with one 10 MV photon beam to generate a 1 × 1 cm^2^ region of 10,000 MU. The container was placed horizontally on the accelerator couch, and the beam entered the container along the shorter axis from the side of the container. The maximal number of 5 consecutive series with no gap between them was obtained. The images obtained were processed with the polyGeVero-CT software package to draw and analyze the profiles in the irradiated and non-irradiated regions (at right angles to the irradiation area). Image uniformity (Std) was analyzed for the VOIs in the center of the image and with the following dimensions: 20 mm diameter and 20 mm length.

### 2.4. Impact of Scanning Time Interval between Consecutvie Scanings on Image Uniformity

The study was performed using the Cathpan^®^ 504 phantom (The Phantom Laboratory, Salem, NY, USA [47]) for the TrueBeam and Halcyon accelerators (CBCT and iCBCT, mode Pelvis and Pelvis Large). Image uniformity measurements were performed in accordance with the installation product acceptance tests (IPA of Varian, Palo Alto, CA, USA) [48]. Measurements were performed at 0 and 5 min intervals for TrueBeam. However, for the Halcyon accelerator, the intervals were 0 and 5 min for the Pelvis mode and 0 and ~6 min for the Pelvis Large mode. The reason behind this was overheating of the X-ray tube. The CTP486 Image uniformity module of the Cathpan^®^ 504 phantom (a cast from a uniform material) was used in this study. VOIs (20 mm diameter, 5 mm length) from different parts of the module (center, left, top, right, bottom) were taken for the calculations of the mean value (calculated with the polyGeVero-CT software package). For example, further calculations performed according to IPA were as follows: Left Image Uniformity = Left − Center.

### 2.5. Impact of Radiation Field Parameters

In this study PABIG^nx^ in the PH-5-DD1 GeVero Co., container (Lodz, Poland) was used. The dosimeter was irradiated with a TrueBeam accelerator. The PABIG^nx^ was placed on the accelerator couch in a horizontal position and was irradiated to observe the impact of: (i) the leaf gap of the high-definition multi-leaf collimator (HD MLC) and (ii) the number of monitor units (MU) on the reading of the polymerized and crosslinked regions in PABIG^nx^ using iCBCT (mode Pelvis, 1 scanning). The following settings were applied: (i) for jaws: 2 × 3 cm^2^, the gap of MLC: 0, 1, 2 and 4 mm, 10 MV FFF photon beam, dose rate 2400 MU/min and 10,000 MU, and (ii) for 2000, 5000 and 10,000 MU, HD MLC gap of 2 mm, 10 MV FFF photon beam and a dose rate of 2400 MU/min.

### 2.6. Impact of Fiducial Markers on Image Quality

A 5% (*w*/*v*) solution of gelatine (type A, 300 Bloom, Sigma-Aldrich, Saint Louis, MO, USA) was prepared in a container (PH-6-DD2, GeVero Co., Lodz, Poland). It should be noted that the container was not intentionally filled with the PABIG^nx^ dosimeter in order to not induce the polymerization and crosslinking of its monomer constituents, which would interfere with the analysis of the marker artifacts.

Four different markers were attached to the container: (i) steel wire, commonly used as a marker in CT (1 mm thickness), (ii) reflective marker spheres (body markers, Brainlab, Munich, Germany), (iii) vitamin E in the shape of round gelatinous capsules (9 mm diameter) and (iv) shielded copper wire of the network cable (copper single-wire conductors with a diameter 0.5 mm). Three markers of each type, one at a time, were attached to the container in a transversal plane (Z = −1 mm) at the 0, 90 and 270° positions.

A container with one type of marker attached was scanned with a TrueBeam medical accelerator (iCBCT, mode: Pelvis). The images obtained after the iCBCT scan were processed with the polyGeVero and polyGeVero-CT software packages (GeVero Co., Lodz, Poland).

### 2.7. Isocenter Determination: 2D and 3D Approaches and Image Processing

PABIG^nx^ in the PH-5-DD1 container (GeVero Co., Lodz, Poland) was used to determine the isocenter for the TrueBeam accelerator using the 2D (star-shot) and 3D approaches. In the case of the 2D approach, the dosimeter was irradiated with four fields (10 MV FFF, 10,000 MU, 2400 MU/min and 2 mm HD MLC leaf gap; gantry angles: 0°, 90°, 150° and 240°). Then, it was scanned with iCBCT, mode: Pelvis (1 series). In addition, the following was examined for the 2D approach: (i) the averaging of iCBCT images; (ii) background subtraction (subtracting non-irradiated PABIG^nx^ images from the irradiated ones); and (iii) the image filtering effect (Mean, Median, Adaptive Mean, Adaptive Median, Kuwahara Mask, Fast Fourier Transform (12 windows), Savitzky–Golay and Envelop) on the determination of the isocenter.

In the case of the 3D approach, the dosimeter was irradiated (10 MV FFF, 10,000 MU, 2400 MU/min, TrueBeam accelerator) with the following settings: a field (HD MLC) of 5 × 5 mm^2^, jaws set to 2 × 2 cm^2^ and the following settings of the gantry, collimator and couch: 0°, 0° and 0°; 90°, 90° and 90°; 150°, 30° and 45°; and 300°, 300° and 300°, respectively. Markers of the shielded copper wire of the network cable were affixed to the PH-5-DD1 container. In both cases (2D and 3D), the container was placed in the accelerator isocenter by using the LAP laser system (LAP GmbH Laser Applikationen, Lüneburg, Germany). Following the irradiation, PABIG^nx^ was scanned with iCBCT, mode: Pelvis (3 series).

### 2.8. The Isocenter Data Processing

The isocenter data processing, after scanning the irradiated PABIG^nx^ 3D polymer gel dosimeter, was performed with the polyGeVero-CT software package (v.1.2, GeVero Co., Poland). A description of the software package v. 1.0.1 can be found elsewhere [40]. The current version of the software package (v.1.2) was enriched with several algorithms, including isocenter data processing related to the 3D polymer gel dosimetry measurements with CT. It should be noted that the polyGeVero-CT software package is compatible with the polyGeVero software package [34,43], which means that the isocenter can also be calculated with the polyGeVero-CT for 3D radiochromic gel and plastic dosimetry after scanning with optical computed tomography (Vista scanner, Modus Medical Devices, London, ON, Canada) and magnetic resonance imaging (3D polymer gel dosimeters), which requires data pre-processing with the polyGeVero software package and then further calculations using polyGeVero-CT. The elaborated isocenter functionality in polyGeVero-CT is based on a calculation approach presented elsewhere [9,10,11]. The isocenter (3D option) was also determined in MPC (Machine Performance Check, Varian, Palo Alto, CA, USA) using the IsoCal geometric phantom. It was compared with the isocenter determined with PABIG^nx^.

## 3. Results and Discussion

### 3.1. Impact of Reconstruction Algorithms and Modes on Image Noise

The impact of the reconstruction algorithms (CBCT and iCBCT) and modes on image noise are reported for the Halcyon (Table 3) and TrueBeam (Table 4) accelerators, which were used to scan PABIG^nx^ in a PH-5-DD1 GeVero Co. container.

In general, both the reconstruction algorithm and mode significantly affected the image noise. iCBCT outperformed CBCT, and a smaller standard deviation calculated for the VOIs was obtained for iCBCT. Pelvis modes (Pelvis, Pelvis Fast, Pelvis Large and Pelvis Large Fast) produced images of low Std for VOIs compared to the other modes used to scan PABIG^nx^ in the PH-5-DD1 GeVero Co. container with the Halcyon accelerator (Table 3). In this case, the Pelvis Large Fast for iCBCT produced the lowest Std for VOI. Similar results were obtained for the scanning of PABIG^nx^ in the PH-5-DD1 container with the TrueBeam accelerator (Table 4). In this case, the iCBCT Pelvis mode gave the lowest Std for the measured VOI.

### 3.2. Impact of Signal Averaging for Reconstruction Algorithms and Selected Modes on Image Quality

**Figure 1:**
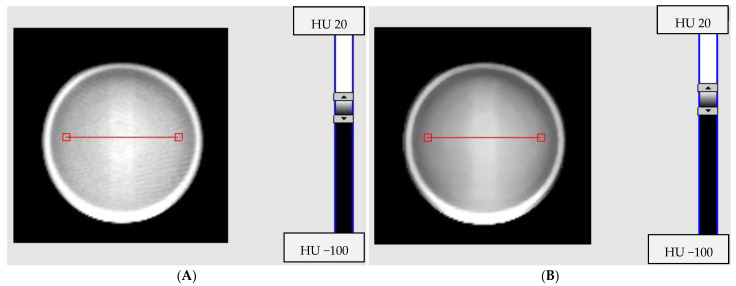

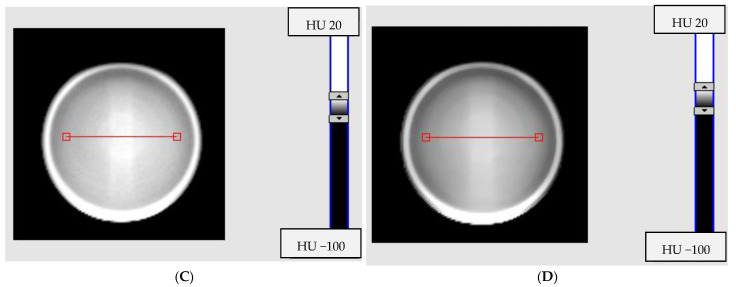
Two-dimensional images (a transversal plane, Z = −1 mm) of irradiated PABIG^nx^ as measured with CBCT and iCBCT (TrueBeam accelerator, Varian). The dosimeter in the PH-6-DD2 container (GeVero Co.): (**A**) is for the CBCT scan, 1 series, (**B**) is for the iCBCT scan, 1 series, (**C**) is for the CBCT with a mean of 5 series and (**D**) is for the iCBCT with a mean of 5 series. Pelvis mode was used for scanning. The red lines in (**A**)−(**D**) indicate the position of profiles presented in Figure 2.

**Figure 2:**
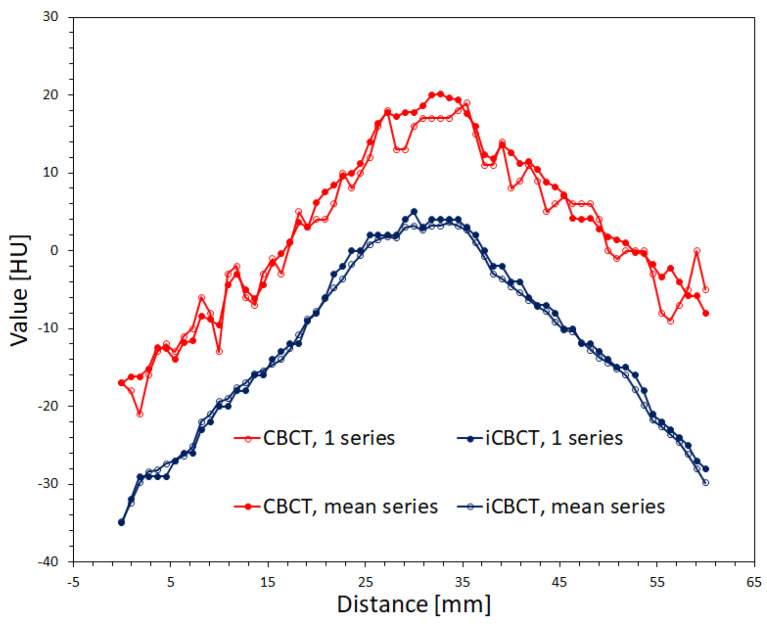
Profiles (as indicated in Figure 1) across the irradiated PABIG^nx^ in the PH-5-DD2 GeVero Co. container (1 × 1 cm^2^, 10,000 MU) for a sample scanned with the TrueBeam accelerator equipped with CBCT and iCBCT. The results are shown for 1 and 1–5 series as the mean.

TrueBeam (CBCT and iCBCT) was used to scan irradiated PABIG^nx^ in a PH-5-DD2 GeVero Co. container (Table 5, Figure 1 and Figure 2) in order to examine the impact of image averaging on image quality (noise for VOIs). The Pelvis and Pelvis Large mode were used. The obtained results again proved superiority of iCBCT over CBCT. The iCBCT Pelvis mode resulted in the lowest Std for the measured VOI.

Averaging the results over five series slightly lowered Std for CBCT; however, it did not improve it substantially for iCBCT (Table 5, Figure 1 and Figure 2). This means that signal averaging for iCBCT is not a must, as it did not markedly improve image uniformity. One scan can be enough for iCBCT, which is advantageous in terms of reducing the overall scan time.

### 3.3. Impact of the Scanning Time Interval between Consecutvie Scanings on Image Uniformity

Image uniformity was examined by using the Cathpan^®^ 504 phantom with the TrueBeam (Table 6) and Halcyon accelerators (Table 7). The criterion of image uniformity was ±30 HU, i.e., if the calculated image uniformity was ≤±30 HU (e.g., signal of VOI for Left, Top, Right or Bottom minus Center), the image obtained for a certain scan protocol was treated as uniform. Otherwise, it was treated as non-uniform. The obtained results (Table 6 and Table 7) showed image uniformity values lower than ±30 HU. This means that scanning with CBCT and iCBCT for the Pelvis and Pelvis Large modes and one to five scans with a gap of 0 and 5 min delivers uniform images according to the selected criterion.

Moreover, no significant effect of the time interval between scans on image homogeneity was observed for the experiment with the TrueBeam and Halcyon accelerators. It was possible to perform up to five consecutive scans for the TrueBeam accelerator with no gap between scans. However, it was different for the Halcyon accelerator, for which it was possible to perform only two consecutive scans with no gap between them. The reason behind this was overheating of the X-ray tube. For the Pelvis mode, a 30% increase in the X-ray tube temperature was observed, which was lower than that for the Pelvis Large mode (46%). Scanning was possible up to an 88% increase in temperature of the X-ray tube. The tube cooled to 22% after 15 min and to 15% after 25 min. Therefore, the gap for scan with the Pelvis mode chosen had to be 5 min and for Pelvis Large, ~6 min. This means that if series averaging from over two series is considered (e.g., for a 3D polymer gel dosimeter), a gap between scans should be applied for the Halcyon accelerator, which is not necessary for the TrueBeam accelerator.

### 3.4. Impact of Radiation Field Parameters

**Figure 3:**
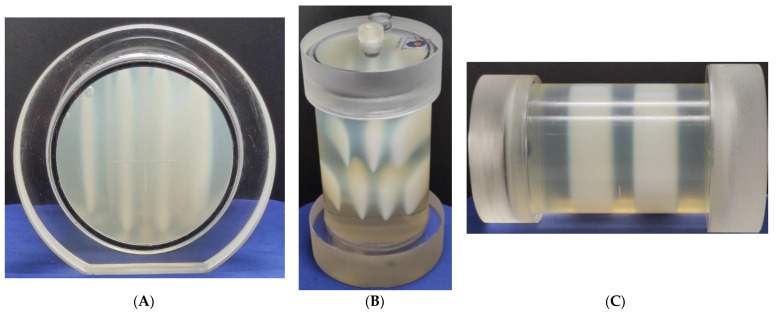

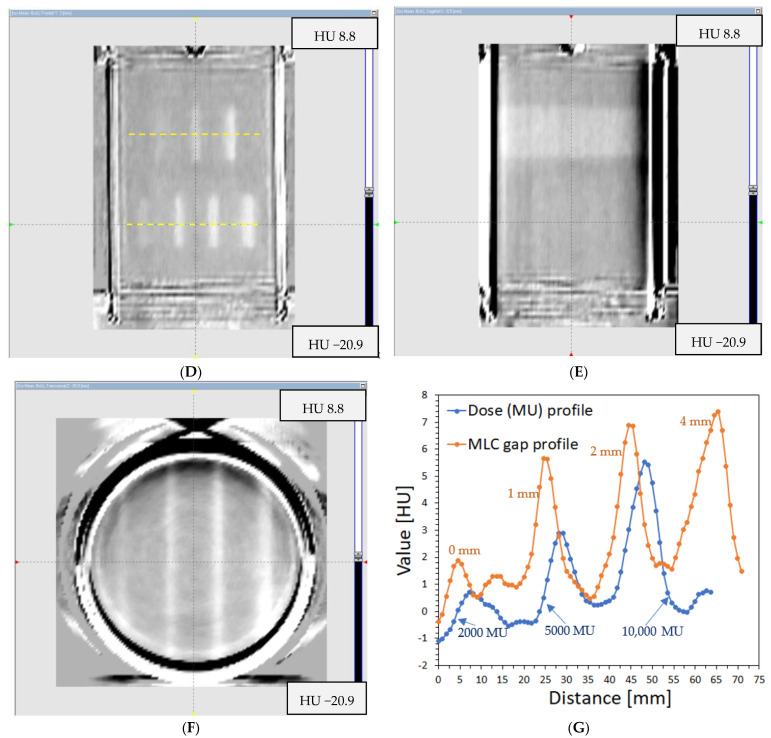
Selection of the radiation field parameters: multi-leaf collimator (MLC) gap and dose (MU) for the determination of the isocenter. The study was performed for PABIG^nx^ in a PH-6-DD2 container (GeVero Co.). In (**A**–**C**), there are photographs of the dosimeter after irradiation; (**A**) is a bottom view in a horizontal position, (**B**) is a front view of the container in a vertical position and (**C**) is a side view of the container in a horizontal position. The irradiated regions (**B**) correspond to different gaps of MLC (closer to the bottom of the container in (**B**)): 0, 1, 2 and 4 mm (for 10,000 MU), from left to right, and they correspond to 2000, 5000 and 10,000 MU, from left to right (closer to the top of the container in (**B**)). (**D**–**F**) are CT images (iCBCT, TrueBeam, Pelvis): for frontal Y: 3 mm; sagittal X: −0.5 mm; and transversal Z: 23.4 mm, respectively. In (**G**), profiles across the irradiated regions, corresponding to the irradiated regions for different gaps of MLC and MU, are presented. The yellow dash lines in (**D**) indicate the positions of the profiles in (**G**). The profiles were smoothed by applying a mean filter (Kernel mode: 2D, Kernel unit: pixels and Kernel size 5 and 3 for Dose (MU) profile and MLC gap profile, respectively).

PABIG^nx^ in the PH-6-DD2 container was used to establish the optimal radiation field parameters to determine the isocenter. Two settings were tested for TrueBeam accelerator: MLC gap and dose (MU). The results obtained are shown in Figure 3. In Figure 3A–C, the white zones correspond to the polymerization and crosslinking of PABIG^nx^ monomeric components, which were induced by ionizing radiation. In Figure 3B, the lower line of the zones corresponds to the regions of different MLC gaps, and the higher line of zones corresponds to different doses (MU). Regarding the gaps, it should be noted that the zone is visible even for a gap equal to 0 mm (first from the left). In Figure 3D–F, iCBCT images are presented for the irradiated dosimeter. In Figure 3G, profiles across the irradiated regions are shown for both different gaps and doses. The main conclusions stemming from these results are as follows: (i) an MLC gap of 2 mm and (ii) a dose of 10,000 MU are suitable for irradiating PABIG^nx^ for isocenter determination (iCBCT, Pelvis mode). This relatively large number of MUs makes the changes in PABIG^nx^ clearly visible in the iCBCT image shortly after irradiation. When determining the isocenter, the user should consider such a large dose to observe a strong signal for the irradiated areas just after irradiation and should scan the dosimeter after irradiation without waiting for an ongoing post-effect in the dosimeter (usually found in 3D polymer gel dosimeters) [38,41] to increase the signal from the irradiated regions. It is assumed that, in the case of MR-guided radiotherapy, the dose and MLC gap selected may be reduced (not examined in this work), as it is commonly known that magnetic resonance imaging (MR imaging) can produce relatively higher signal-to-noise images of irradiated 3D polymer gel dosimeters. Thus, the detection of lower dose regions is feasible [41,42,43].

### 3.5. Impact of Fiducial Markers on Image Quality

**Figure 4:**
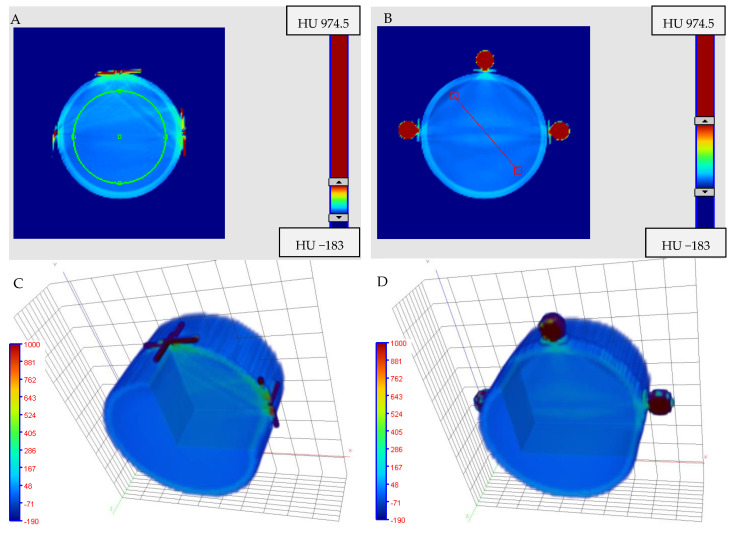

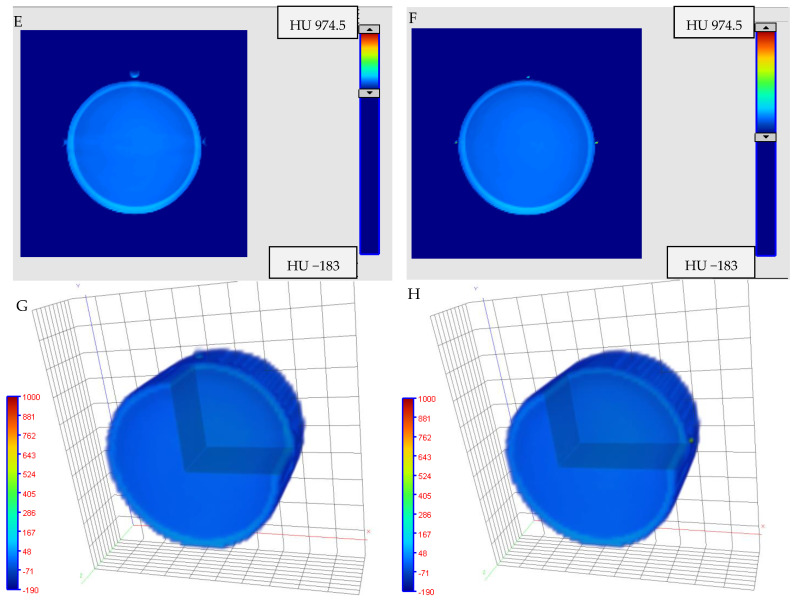
Two-dimensional (transversal plane, Z = −1 mm) and three-dimensional images of PH-6-DD2 container (GeVero Co.) with 5% gelatine (iCBCT, Pelvis, TrueBeam accelerator, Varian). The following markers were attached to the container at 0, 90 and 270°: steel wire commonly used as a marker in CT (1 mm thickness) (**A**,**C**), reflective marker spheres (Brainlab) (**B**,**D**), vitamin (**E**) of the shape of round capsules (**E**,**G**) and the shielded copper wire of the network cable (0.5 mm thickness) (**F**,**H**). The green circle in (**A**) indicates a cylindrical VOI of 32.8 mm in diameter and 2 mm long, applied to each scanned container with different markers. The red line in (**B**) is the position of the profiles applied to each scanned sample (see Figure 5). iCBCT images were processed with the polyGeVero-CT and polyGeVero (GeVero Co.) software packages.

**Figure 5:**
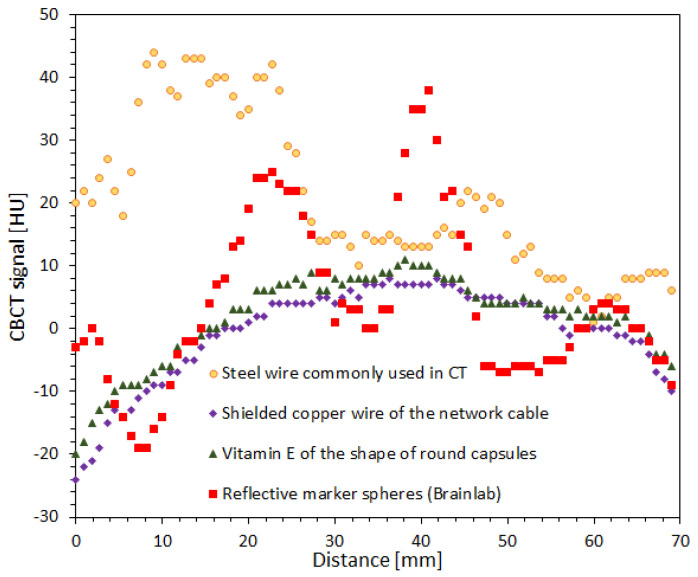
Comparison of profiles randomly drawn across each scanned container with different markers, as indicated in Figure 4B.

Four different markers were used to study their impact on image quality in cone-beam computed tomography (CBCT, TrueBeam accelerator) scanning. The results of this experiment are presented in Figure 4 and Figure 5. In Figure 4, 2D and 3D images of a PH-6-DD 2 container (GeVero Co.) with 5% gelatine and attached markers are presented. The 2D images are the transversal planes through the center of the markers with the most visible marker artifacts. The 3D images illustrate the entire part of the container with attached markers. In this case, the cutting plane option of the polyGeVero software package was applied to show the interior of the 3D images and related markers artifacts. Note that the visibility of the markers is related to their size and composition with respect to the scanning parameters of the 3D scanner. Those made of steel wire commonly used as a marker in CT (Figure 4A,C) and reflective marker spheres (Brainlab) (Figure 4B,D) are well visible due to their size and composition. The markers made of vitamin E capsules are much less visible (Figure 4E,G) despite the fact that they are relatively large with a diameter of 9 mm. Finally, the markers of the shielded copper wire of the network cable (Figure 4F,H) are the least visible in the 3D image due to their small size; however, they are clearly visible as dots of a bright intensity in 2D images (Figure 4F). The naked eye inspection of 2D and 3D images in different color palette ranges revealed strong artifacts for both markers made of steel wire commonly used as a marker in CT (Figure 4A,C) and the reflective marker spheres (Brainlab) (Figure 4B,D). Much fainter artifacts can be discerned, upon close examination, for markers made of vitamin E capsules (Figure 4E,G). However, 2D and 3D images with markers made of shielded copper wire of the network cable seemed to be devoid of marker artifacts.

After organoleptic inspection, the mean values of VOIs, as indicated in Figure 4A, were calculated for all samples with four different markers, as follows: (i) 17.9 ± 18.81 HU (Figure 4A), (ii) 1.8 ± 10.75 HU (Figure 4B), (iii) −0.3 ± 6 HU (Figure 4E) and (iv) −2.5 ± 6.27 HU (Figure 4F). These values mean that the highest non-uniformity of images (high Std) are for two types of markers: steel wire commonly used as a marker in CT and reflective marker spheres (Brainlab). The lowest non-uniformity of images (low Std) is for vitamin E capsules and shielded copper wire of the network cable.

Profiles across the 2D images were obtained as indicated in Figure 4B in order to further investigate the non-uniformity of the image from the markers attached to the gelatine container. The results are presented in Figure 5. The artifacts from steel wire commonly used as a marker in CT and reflective marker spheres (Brainlab) are the reasons for the wavy profiles and large fluctuations in the CBCT signal (HU). In turn, both the vitamin E capsules and the shielded copper wire of the network cable gave low noise profiles with an inverted parabolic shape. The reason for this profile shape is the CBCT scanning conditions of the container. The container was scanned on an accelerator bench in air, not in a water phantom (consult Figure 4 elsewhere [40]; the shape of the profiles is related to the CBCT X-ray beam hardening effect, as discussed elsewhere [49,50]). In summary, of the four markers used, two are not applicable to isocenter studies: steel wire commonly used as a marker in CT and reflective marker spheres (Brainlab). The artifacts caused by these markers burden the CBCT images and can significantly interfere with signals recorded by a 3D polymer gel dosimeter after X-ray irradiation during isocenter determination. The other two markers are feasible for isocenter determination. It seems, however, that the marker made of shielded copper wire of the network cable outperforms the vitamin E marker due to the slightly lower noise level of the profile (Figure 5), much smaller size and strong signal in CBCT images, which enable the drawing of intersecting lines passing through the markers with high precision.

### 3.6. Isocenter Determination: 2D and 3D Approaches and Image Processing

#### 3.6.1. Impact of Image Processing

It was shown elsewhere for spiral CT measurements of 3D polymer gel dosimeters [40] that CT image processing significantly affects image quality. Proper image processing can reduce image noise and bring out the details of the image accordingly. The best results for the spiral CT of the 3D polymer gel dosimeter were obtained by averaging the series of 20 images, subtracting the background (non-irradiated sample) and filtering using the mean filter (3 × 3 mm^2^, 3D calculation mode, 1 iteration), followed by a Nutall window (25 iterations) for 3D polymer gel dosimeters irradiated and scanned in a water phantom [40]. However, the current study was performed using medical accelerators combined with CBCT and iCBCT in a single device as a scanning option for 3D polymer gel dosimeters, and it required the imaging procedure for these instruments to be investigated to shorten the overall time for isocenter determination. Therefore, the dosimeters were irradiated and scanned in the air (not in the water phantom), and one dosimeter served both as the background to be scanned and for the irradiation (after scanning the background) according to the isocenter pattern.

**Figure 6:**
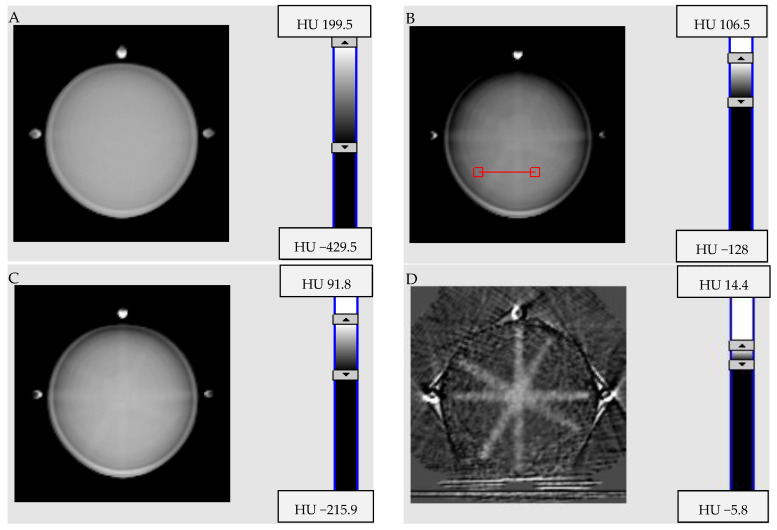

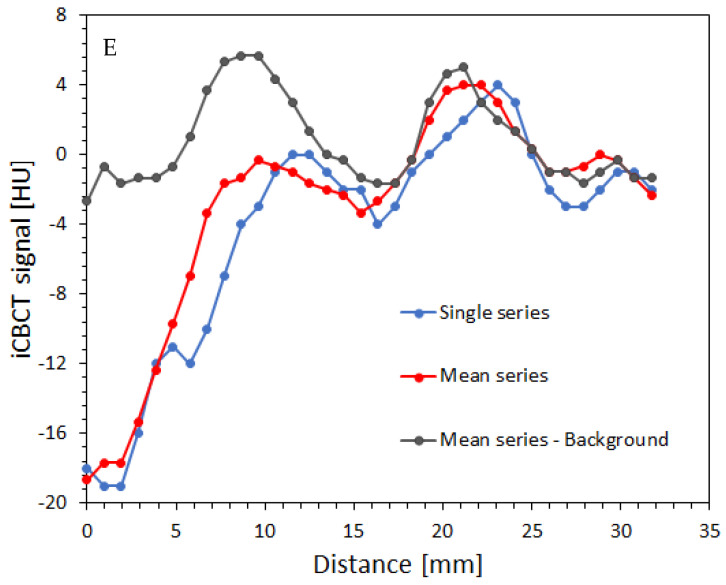
Processing of iCBCT images of PABIG^nx^ in PH-5-DD1 GeVero Co. container irradiated with TrueBeam accelerator (mode: Pelvis): (**A**) is for the background (non-irradiated dosimeter), (**B**) is for one series, (**C**) is for the mean series calculated from three series, (**D**) is the image of (**C**) with the background subtracted (**A**–**C**), and (**E**) shows the profiles for (**B**–**D**). The HU scales have been set to best visualize the changes inside the container. The red line in (**B**) shows the position of the profiles.

The results obtained for the background, single series, mean series (from three series) and mean series with the background subtracted are shown in Figure 6. A detailed analysis of the images yielded the following conclusions. Single-series images (Figure 6B) make it possible to distinguish the pattern of four beams intersecting in the center of the image. The mean series represents slightly smoothed images (Figure 6C,E). Subtracting the background from the mean series reveals a clear and easily discernible pattern of four intersecting isocenter beams (Figure 6D). This is in line with the background subtraction results reported for the NIPAM dosimeter used for the isocenter determination (CBCT) [38]. Therefore, the main conclusion is that the images of the PABIG^nx^ 3D polymer gel dosimeter after scanning can be averaged over three series, from which the background (images of non-irradiated PABIG^nx^) should be subtracted. 

Background subtraction has been shown to be an important operation of extracting the irradiation pattern from an image of an irradiated dosimeter to determine the isocenter. Apart from that, the filtering of the images was tested to examine the possibility of improving the quality of the intersecting beam pattern in the images. It should be noted that polyGeVero-CT comes with numerous filtering options such as Mean, Median, Adaptive Mean, Adaptive Median, Kuwahara Mask, Fast Fourier Transform (12 windows), Savitzky–Golay and Envelop. These can be examined while processing any CT images of 3D polymer gel dosimeters. The common purpose of filtering the isocenter images is to amplify the signal from the irradiated regions of the 3D dosimeter and to reduce the image noise to facilitate the polyGeVero-CT ioscenter option to automatically perform the isocenter calculation.

After testing various filters, the following conclusions were drawn: (i) The Mean, Adaptive Mean and Envelope filters seem to find some application in reducing PABIG^nx^ isocenter image noise. (ii) Filtering should include both background (non-irradiated) and irradiated images; then, the background can be subtracted from the irradiated dosimeter image. (iii) Subtracting the non-filtered background from the filtered irradiated dosimeter image may leave ring artifacts on the processed image that can interfere with the automatic isocenter determination by the Isocenter option of the polyGeVero-CT software package.

**Figure 7:**
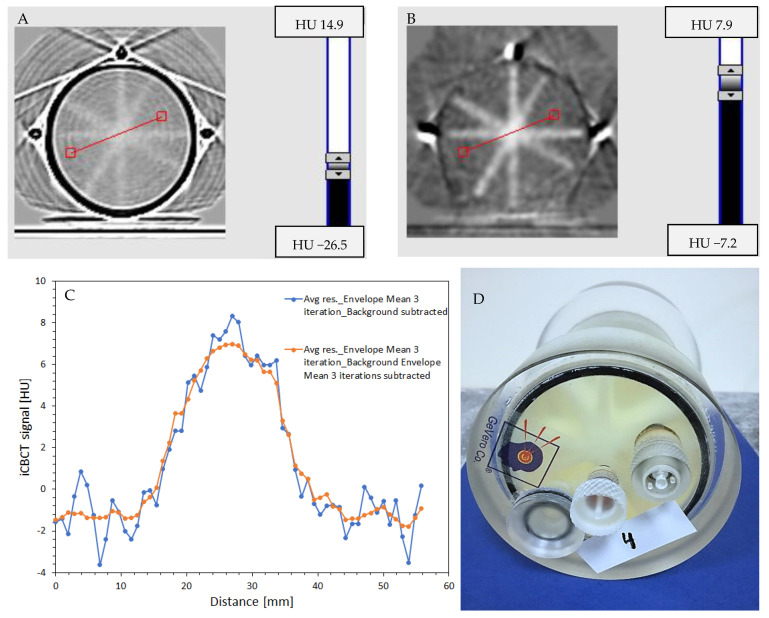
Processing of iCBCT images of PABIG^nx^ in PH-5-DD1 GeVero Co. container irradiated with TrueBeam accelerator (mode: Pelvis): (**A**) is for the mean series (calculated from three series) filtered with the Envelope filter (mean, 3 iterations, Kernel = 3, Kernel mode = 2D, Kernel unit = pixels), with background subtracted (non-irradiated dosimeter), and (**B**) is like (**A**). However, the background was also filtered with the Envelope filter (same parameters). (**C**) is for profiles along the red lines in (**A**,**B**). (**D**) is for a photograph of PABIG^nx^; the white regions inside the container are the effect of the polymerization and crosslinking of PABIG^nx^ monomers and correspond to the photon beams passing through the dosimeter according to the star-shot isocenter pattern of irradiation.

The exemplary results of the effect of applying the Envelope filter on the mean series following background subtraction are shown in Figure 7. Two approaches to background subtraction were tested for the background filtered with the Envelope filter and the non-filtered background image. The inspection of the profiles and acquired images (Figure 7A–C) revealed that subtracting the non-filtered background from the Envelope-filtered mean series keeps ring artifacts (Figure 7A), as mentioned above, which may make isocenter determination more difficult. It is therefore advisable to subtract the filtered background, which leads to a smooth image and reduced noise profiles (Figure 7B,C).

The optimal procedures and parameters for determining the irradiation isocenter for the PABIG^nx^, and Halcyon and TrueBeam accelerators are summarized in Table 8.

#### 3.6.2. Isocenter Calculation: 2D approach

**Figure 8:**
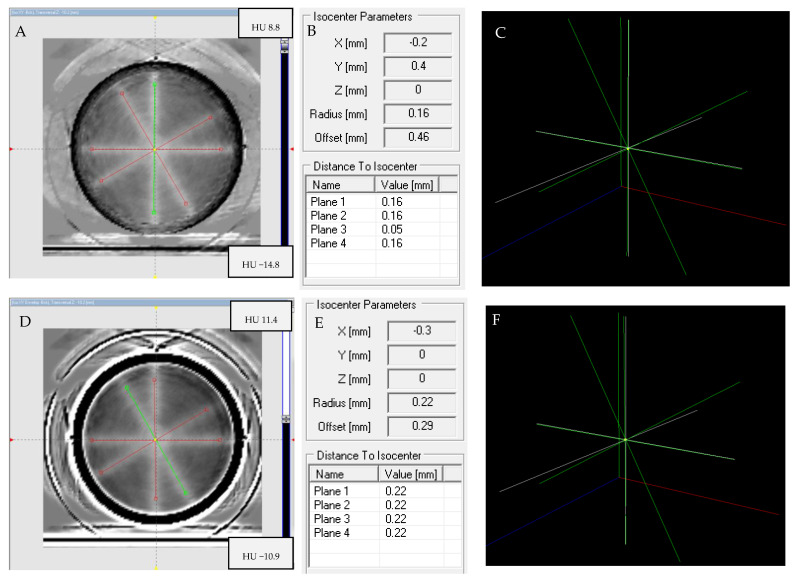

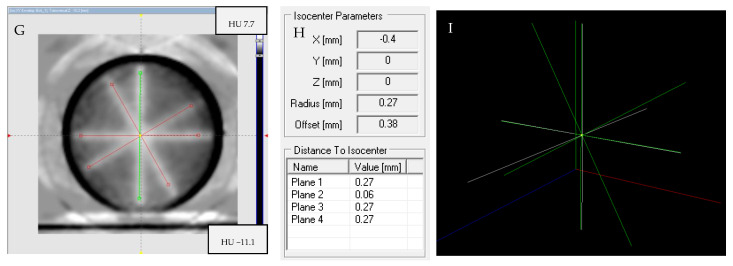
Isocenter determination. Processing of iCBCT images of PABIG^nx^ in PH-5-DD1 GeVero Co. container irradiated with the TrueBeam accelerator (mode: Pelvis): (**A**) is for the mean series (calculated from three series) with the mean background (calculated also from three series) subtracted; (**B**) corresponds to the isocenter results of (**A**), and (**C**) is for the 3D visualization of the isocenter determined in (**A**); (**D**) is for the mean series (calculated from three series) filtered with the Envelope filter (mean, 3 iterations, Kernel = 3, Kernel mode = 2D, Kernel unit = pixels) with the mean background subtracted (calculated from three series); (**E**) corresponds to the isocenter results, and (**F**) is for the 3D visualization of the isocenter determined in (**D**); (**G**) is like (**D**), however, the background was also filtered with the Envelope filter (same parameters); (**H**) is for the isocenter results for (**G**), and (**I**) is the 3D visualization of the isocenter determined in (**G**). The colored lines in (**A**,**D**,**G**) intersect at the isocenter. The isocenter is marked with a yellow dot.

The iCBCT images of the star-shot irradiated PABIG^nx^, processed according to the following pattern, were used for the isocenter determination: (i) mean series obtained from three series with the mean background (also from three series) subtracted (Figure 8A–C); (ii) mean series obtained from three series filtered with the Envelope filter (mean, 3 iterations, Kernel = 3, Kernel mode = 2D, Kernel unit = pixels) with the non-filtered background subtracted (Figure 8D–F); and (iii) mean series obtained from three series filtered with the Envelope filter (mean, 3 iterations, Kernel = 3, Kernel mode = 2D, Kernel unit = pixels) with the Envelope filtered background (same parameters as the irradiated sample) subtracted (Figure 8G–I). The star shot 2D (XY) plane of polyGeVero-CT was applied to determine the isocenter for the irradiated PABIG^nx^ with respect to the selected Center option (as Offset), which is the viewing plane intersection passing through three fiducial markers (shielded copper wire of the network cable). The obtained isocenter results (isocenter circle radius) in Figure 8B,E,H are within the tolerance level for the star shot measurement; ±1 mm, according to the Task Group 142 report [51]. From a practical point of view, image filtering did not improve the determination of the isocenter in this particular study. Subtracting the non-filtered background from the Envelope-filtered images revealed ring artifacts (Figure 8D). Subtracting the Envelope-filtered background the same way from the filtered image of the irradiated PABIG^nx^ slightly blurred the ring artifacts. In summary, it seems that limiting image operations to averaging from three series following background subtraction is sufficient to determine the isocenter using PABIG^nx^ scanned with iCBCT TrueBeam (Pelvis). 

#### 3.6.3. Isocenter Calculation: 3D Approach

**Figure 9:**
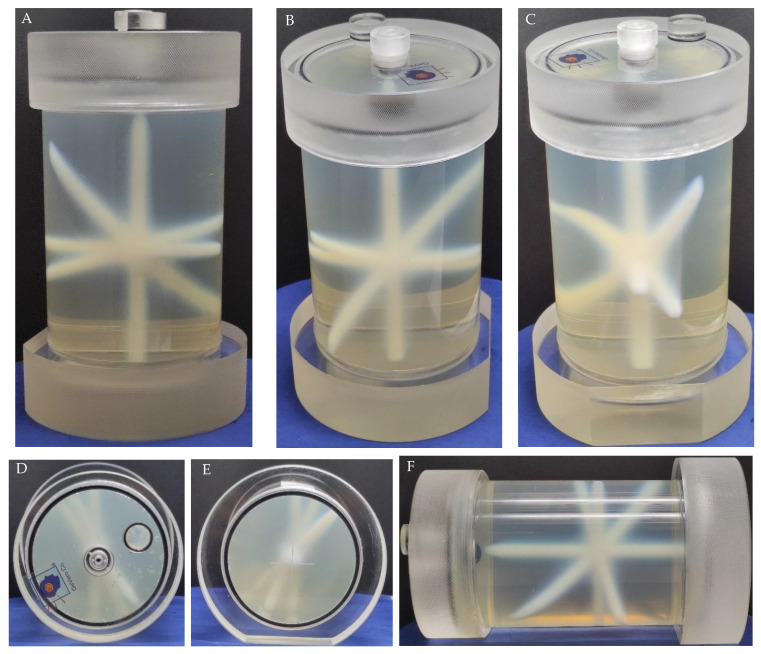
Isocenter determination: 3D option. PABIG^nx^ in PH-5-DD1 GeVero Co. container was irradiated (**A**–**F**) and scanned with a TrueBeam accelerator (iCBCT, mode: Pelvis, mean over 3 series). The photographs in (**A**–**C**) are for the rotated sample ((**A**): 0°, (**B**): 180° and (**C**): 270°) to better inspect of the irradiation pattern; (**D**,**E**) are the top and bottom views, respectively, and (**F**) is the side view.

**Figure 10:**
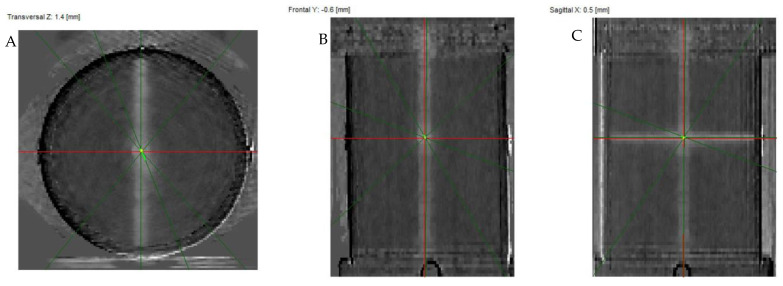

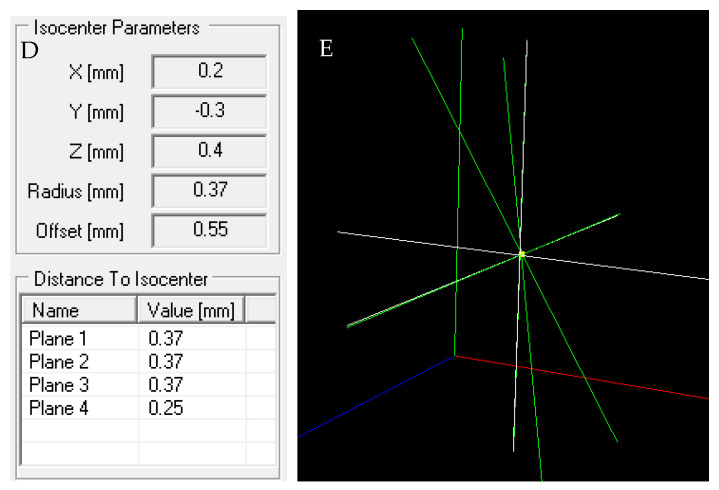
Isocenter determination: 3D option. PABIG^nx^ in PH-5-DD1 GeVero Co. container was irradiated and scanned with the TrueBeam accelerator (iCBCT, mode: Pelvis, mean over 3 series; background (mean over 3 series) subtracted from the scans of the irradiated sample), and an isocenter was determined using the polyGeVero-CT software package (GeVero Co.). (**A**) is for transversal Z plane, (**B**) is for frontal Y plane, (**C**) is for sagittal X plane, (**D**) is a table with the results of the isocenter determination and (**E**) is for the 3D view of the isocenter determination; the yellow dot in the center corresponds to the isocenter.

PABIG^nx^ in the PH-5-DD1 container was irradiated according to the 3D irradiation pattern to verify the TrueBeam accelerator isocenter. The results are shown in Figure 9 and Figure 10. In Figure 9A–F, photographs of the PABIG^nx^ container are shown to better inspect the irradiation pattern with the naked eye. The white regions corresponding to the polymerization and crosslinking of the PABIG^nx^ monomeric ingredients are clearly seen to cross in a point. The dosimeter was scanned immediately after irradiation (iCBCT, Pelvis), and the results of the scanning and data processing are shown in Figure 10. The isocenter was determined to have a radius of 0.37 mm and an offset of 0.55 mm. The isocenter determined in MPC (Machine Performance Check) using the IsoCal geometric phantom is equal to 0.35 mm (the threshold is ±0.5 mm).

The results presented in Section 3.6.2 and Section 3.6.3 illustrate the robustness of the reported tool for the determination of the accelerators’ isocenters, with PABIG^nx^ in the PH-5-DD1 container in conjunction with the polyGeVero-CT software package and iCBCT (Pelvis) scanning.

### 3.7. Time and Effort to Determine the Isocenter

The sum of the operations required to determine the irradiation isocenter includes: (i) the preparation of the PABIG^nx^ dosimeter in a dedicated, pre-prepared container; (ii) the irradiation of PABIG^nx^; (iii) the scanning of irradiated PABIG^nx^; and (iv) the calculation of the results. PABIG^nx^ is not a complicated dosimeter for manufacturing. Its preparation is relatively easy to perform due to the low concentrations of the main ingredients and the reduced number of ingredients to mix. The time required to prepare PABIG^nx^ is primarily determined by cooling the composition prior to adding the PEGDA monomer. In general, manufacturing (1 L dosimeter) may only take about 2 h with enhanced cooling or several hours if cooling is performed at room temperature. Nevertheless, the dosimeter is ready for irradiation after about 24 h after preparation due to the solidification of gelatine at room temperature. The irradiation of PABIG^nx^ takes place immediately on a couch of an accelerator. This differs from the irradiation of a dosimeter in a water phantom, which was reported in another study with a CT readout [40]. In the case of determining the irradiation isocenter, the irradiation (and scanning) was simplified by not using a water phantom.

The time required to irradiate PABIG^nx^ according to the 2D and 3D patterns (10 MV FFF, 10,000 MU, 2400 MU/min, TrueBeam) is 5–6 min per beam (including set up and irradiation). The scanning of irradiated PABIG^nx^ is performed with the same instrument, the TrueBeam (or Halcyon) accelerator using iCBCT (mode: Pelvis, 1–3 scans), and it can be performed immediately after irradiation. It takes approximately 2 min to scan the dosimeter (1 series) independent of the reconstruction, CBCT or iCBCT. The DICOM files obtained after scanning are processed with the commercial software, polyGeVero-CT, having dedicated data processing functionality towards the determination of the irradiation isocenter. The time needed for such calculations (from uploading data to obtaining results) is less than 15 min. Overall, the total time required to determine the irradiation isocenter for the customer is about 1 h, and for a researcher (manufacturer), 24 h (dosimeter preparation) and about 1 h (other steps) are required. The time assessed is close to that reported elsewhere [38] for setup, irradiation and readout (without data processing; not reported in that work).

## 4. Conclusions

This work was aimed at establishing the conditions for determining the irradiation isocenter for two accelerators: TrueBeam and Halcyon, using 3D polymer gel dosimetry, and it presents exemplary isocenter determination. The following has been established. PABIG^nx^ in a dedicated ~1 L container (PH-5-DD1, GeVero Co.) is suitable for isocenter determination (2D and 3D approach). Fiducial markers made of a shielded copper wire of the network cable attached to the container are suitable, as they do not produce artifacts that affect the quality of the CT image. To irradiate PABIG^nx^, it should be placed immediately on an accelerator couch (no water phantom is required). The radiation field parameters, with a multi-leaf collimator (MLC) gap of 2 mm and a dose of 10,000 MU, should be chosen. Scanning should be performed both for the non-irradiated and irradiated dosimeter. The scanning of the irradiated dosimeter can be performed immediately after irradiation. The scan should be performed for the iCBCT reconstruction algorithm and the Plevis mode for the TrueBeam accelerator and iCBCT and Pelvis or Pelvis Large Fast for the Halcyon accelerator. One scan is enough to obtain proper results for iCBCT (three scans can be performed for mean series calculation). Consecutive scanning with 0 min gap between scans can be performed for the TrueBeam accelerator (five scans); however, a maximum of two scans can be performed for the Halcyon accelerator due to overheating of the X-ray tube. Image processing with the polyGeVero-CT software package includes: (i) one or mean series calculations from three series—both background and irradiated PABIG^nx^; (ii) subtracting the background series from the series for irradiated dosimeter and (iii) calculating the irradiation isocenter. No image filtration is obligatory to determine the isocenter according to this study for PABIG^nx^.

## Figures and Tables

**Table 1:** CBCT and iCBCT acquisition parameters for the Halcyon accelerator [46]; CTDI_Vol_ denotes volume computed tomography dose index, and DLP means dose length product.

Mode	Reconstruction	Energy [kV]	Exposure [mAs]	CTDI_Vol_ [mGy]	DLP [mGy·cm]	Acquisition Time [s]	Scan Diameter [cm]
Image Gently	CBCT, iCBCT	80	84	1.08	16.3	16.6	28.2
Image Gently Large	CBCT, iCBCT	100	90	2.38	35.6	16.6	38.4
Head	CBCT, iCBCT	100	126	3.33	49.9	16.6	28.2
Head Low Dose	CBCT, iCBCT	100	42	1.11	16.6	16.6	28.2
Breast	CBCT	125	45	0.90	13.5	16.6	49.2
Thorax Fast	CBCT	125	270	5.4	81	16.6	49.2
Pelvis Fast	CBCT, iCBCT	125	560	11.2	168	21.2	49.2
Thorax	CBCT	125	294	5.88	88.2	30.8	49.2
Pelvis	CBCT, iCBCT	125	1080	21.6	324	36.7	49.2
Pelvis Large Fast	CBCT, iCBCT	140	672	17.74	266.1	25	49.2
Pelvis Large	CBCT, iCBCT	140	1440	38.02	570.2	40.6	49.2

**Table 2:** CBCT and iCBCT acquisition parameters (extracted from the instrument) for the TrueBeam accelerator (slice thickness = 2 mm; matrix size = 512 pixels; filter: S—Standard, A—Auto, Sm—Smooth).

Mode	Reconstruction	Energy [kV]	Exposure [mAs]	CTDI_Vol_ [mGy]	DLP [mGy·cm]	Fan Type	Acquisition Time [s]	Filter	Noise Suppression
Head	iCBCT	100	150	3.17	67.7	Full	33	S	Medium
Head	CBCT	100	150	3.17		Full	33	A	-
Image Gently	iCBCT	80	100	0.94	20.1	Full	33	S	Medium
Image Gently	CBCT	80	100	0.94		Full	33	A	-
Pelvis	iCBCT	125	1080	15.98	342.1	Half	60	S	Medium
Pelvis	iCBCT Fast	125	1080	15.98		Half	60	S	Medium
Pelvis	CBCT	125	1080	15.98		Half	60	A	-
Pelvis Large	iCBCT	140	1688	36.79	787.3	Half	60	S	Medium
Pelvis Large	iCBCT Fast	140	1688	36.79		Half	60	S	Medium
Pelvis Large	CBCT	140	1688	36.79		Half	60	A	-
Short Thorax	CBCT	125	210	3.44	73.7	Full	23	Sm	-
Spotlight	CBCT	125	750	12.30	263.2	Full	33	Sm	-
Thorax	CBCT	125	270	4.0	85.5	Half	60	A	-

**Table 3:** Impact of reconstruction algorithms and modes on image uniformity for scanning PABIG^nx^ in PH-5-DD1 GeVero Co. container with the Halcyon accelerator equipped with CBCT and iCBCT. VOI: 20 mm diameter, 20 mm height.

Mode	Standard Deviation for VOI [HU]
Cone-beam CT (CBCT)	
Image Gently	25.841
Image Gently Large	16.506
Head	18.917
Head Low Dose	24.452
Pelvis	2.984
Pelvis Fast	7.403
Pelvis Large	2.772
Pelvis Large Fast	4.570
Iterative reconstruction CBCT (iCBCT)	
Image Gently	9.581
Image Gently Large	4.475
Head	3.679
Head Low Dose	8.465
Pelvis	2.129
Pelvis Fast	2.703
Pelvis Large	1.585
Pelvis Large Fast	1.511

**Table 4:** Impact of reconstruction algorithms and modes on image uniformity for scanning PABIG^nx^ in PH-5-DD1 GeVero Co. container with the TrueBeam accelerator equipped with CBCT and iCBCT. VOI: 20 mm diameter, 20 mm height.

Mode	Standard Deviation for VOI [HU]
Cone-beam CT (CBCT)	
Head	11.867
Image Gently	17.916
Pelvis Large	4.986
Pelvis	2.743
Short Thorax	7.269
Spotlight	4.785
Thorax	3.746
Iterative reconstruction CBCT (iCBCT)	
Head	4.115
Image Gently	6.875
Pelvis Large	3.233
Pelvis	1.555

**Table 5:** Impact of reconstruction algorithms and modes on image uniformity for scanning irradiated PABIG^nx^ in PH-6-DD2 GeVero Co. container with the TrueBeam accelerator equipped with CBCT and iCBCT (1 × 1 cm^2^, 10,000 MU). VOI: 20 mm diameter, 20 mm height. The results are shown for 1 and 1–5 series as a mean (s_1 and s_1–s_5).

Mode	Standard Deviation for VOI [HU]
Cone-beam CT (CBCT)	
Pelvis s_1	4.81
Pelvis Large s_1	7.30
Pelvis s_1–s_5	4.53
Pelvis Large s_1–s_5	7.18
Iterative reconstruction CBCT (iCBCT)	
Pelvis s_1	4.19
Pelvis Large s_1	6.58
Pelvis s_1–s_5	4.20
Pelvis Large s_1–s_5	6.64

**Table 6:** Impact of reconstruction algorithms and modes on the image uniformity for scanning the Cathpan^®^ 504 phantom with the TrueBeam accelerator equipped with CBCT and iCBCT. The results are shown for 0 and 5 min gaps between measurements (i_0 and i_5, respectively) and for 1–5 series (s_1–s_5). VOI: 20 mm diameter, 5 mm height.

	Mean Value [HU]					Image Uniformity (±30 HU)					
Mode/Gap (i_0, i_5)/Series (s_1–s_5)	Center	Left	Top	Right	Bottom	Left (1)	Top (2)	Right (3)	Bottom (4)	Mean (1–4)	Mean (Series s_1–s_5)
Cone-beam CT (CBCT)											
Pelvis i_0 s_1	11.58	1.89	3.86	6.36	2.70	−9.69	−7.73	−5.23	−8.88	−7.88	−7.86
Pelvis i_0 s_2	10.61	3.82	4.11	2.93	1.83	−6.79	−6.50	−7.68	−8.78	−7.44	
Pelvis i_0 s_3	11.43	2.33	3.89	5.57	2.80	−9.10	−7.53	−5.85	−8.62	−7.78	
Pelvis i_0 s_4	11.22	3.45	3.93	3.00	1.59	−7.76	−7.29	−8.21	−9.63	−8.22	
Pelvis i_0 s_5	11.49	2.20	3.46	5.48	2.83	−9.29	−8.03	−6.01	−8.66	−8.00	
Pelvis i_5 s_1	13.42	3.28	5.41	7.57	4.01	−10.13	−8.01	−5.84	−9.40	−8.35	−8.02
Pelvis i_5 s_2	12.21	5.29	5.20	3.71	2.88	−6.92	−7.01	−8.50	−9.33	−7.94	
Pelvis i_5 s_3	12.89	3.03	4.92	7.38	3.82	−9.87	−7.97	−5.51	−9.08	−8.11	
Pelvis i_5 s_4	11.72	4.97	5.24	3.41	2.63	−6.75	−6.49	−8.32	−9.10	−7.66	
Pelvis i_5 s_5	12.51	2.41	4.96	6.91	3.55	−10.10	−7.55	−5.60	−8.96	−8.05	
PelvisLarge i_0 s_1	28.42	7.87	7.16	5.87	5.30	−20.55	−21.27	−22.55	−23.12	−21.87	−22.33
PelvisLarge i_0 s_2	30.88	6.77	9.49	11.44	7.46	−24.10	−21.39	−19.44	−23.42	−22.09	
PelvisLarge i_0 s_3	29.77	8.08	8.20	6.94	6.13	−21.69	−21.57	−22.82	−23.64	−22.43	
PelvisLarge i_0 s_4	30.74	6.86	8.67	10.31	6.59	−23.88	−22.07	−20.43	−24.15	−22.63	
PelvisLarge i_0 s_5	28.87	7.09	6.92	6.00	4.96	−21.77	−21.95	−22.87	−23.91	−22.62	
PelvisLarge i_5 s_1	27.00	3.20	5.31	7.50	3.57	−23.80	−21.69	−19.50	−23.43	−22.10	−22.10
PelvisLarge i_5 s_2	25.58	3.76	3.99	2.51	1.86	−21.82	−21.59	−23.07	−23.71	−22.55	
PelvisLarge i_5 s_3	26.25	2.47	4.66	6.23	2.79	−23.78	−21.59	−20.02	−23.46	−22.21	
PelvisLarge i_5 s_4	24.32	3.39	3.11	2.04	0.99	−20.93	−21.22	−22.29	−23.33	−21.94	
PelvisLarge i_5 s_5	24.89	1.73	3.83	5.19	2.00	−23.17	−21.06	−19.70	−22.89	−21.70	
Iterative reconstruction CBCT (iCBCT)											
Pelvis i_0 s_1	−6.73	−9.97	−9.19	−11.17	−11.32	−3.24	−2.46	−4.44	−4.59	−3.68	−4.12
Pelvis i_0 s_2	−5.97	−11.10	−9.39	−9.65	−10.72	−5.13	−3.43	−3.68	−4.75	−4.25	
Pelvis i_0 s_3	−7.00	−11.00	−10.26	−11.84	−12.18	−4.00	−3.26	−4.84	−5.18	−4.32	
Pelvis i_0 s_4	−6.76	−11.64	−10.16	−10.48	−11.31	−4.88	−3.41	−3.73	−4.56	−4.14	
Pelvis i_0 s_5	−7.61	−11.31	−10.72	−12.56	−12.61	−3.70	−3.11	−4.95	−5.00	−4.19	
Pelvis i_5 s_1	−5.27	−8.69	−8.06	−10.00	−10.24	−3.42	−2.79	−4.73	−4.97	−3.98	−4.11
Pelvis i_5 s_2	−4.11	−9.91	−8.03	−7.71	−9.00	−5.80	−3.92	−3.60	−4.89	−4.55	
Pelvis i_5 s_3	−4.88	−8.45	−7.76	−9.85	−10.10	−3.58	−2.88	−4.97	−5.22	−4.16	
Pelvis i_5 s_4	−4.89	−10.24	−8.20	−7.86	−9.52	−5.36	−3.31	−2.97	−4.63	−4.07	
Pelvis i_5 s_5	−5.85	−8.98	−8.57	−10.20	−10.69	−3.13	−2.72	−4.36	−4.84	−3.76	
PelvisLarge i_0 s_1	−0.71	−0.39	−0.27	−2.44	−2.68	0.32	0.45	−1.72	−1.96	−0.73	−2.68
PelvisLarge i_0 s_2	0.36	−0.88	0.13	−1.43	−2.25	−1.24	−0.24	−1.79	−2.62	−1.47	
PelvisLarge i_0 s_3	−2.03	−2.82	−2.33	−4.19	−4.92	−0.78	−0.29	−2.15	−2.89	−1.53	
PelvisLarge i_0 s_4	−0.62	−2.27	−1.37	−3.29	−4.17	−1.65	−0.75	−2.67	−3.54	−2.15	
PelvisLarge i_0 s_5	17.41	10.01	11.51	10.38	7.62	−7.39	−5.90	−7.03	−9.79	−7.53	
PelvisLarge i_5 s_1	−2.01	−3.96	−2.27	−3.91	−5.36	−1.95	−0.26	−1.90	−3.35	−1.87	−2.12
PelvisLarge i_5 s_2	−3.98	−4.73	−4.87	−6.89	−7.21	−0.75	−0.89	−2.91	−3.24	−1.95	
PelvisLarge i_5 s_3	−2.49	−5.10	−3.40	−5.02	−6.42	−2.62	−0.91	−2.53	−3.93	−2.50	
PelvisLarge i_5 s_4	−4.69	−5.94	−5.69	−7.66	−8.24	−1.25	−1.00	−2.97	−3.55	−2.19	
PelvisLarge i_5 s_5	−3.36	−5.42	−3.78	−5.80	−6.79	−2.07	−0.42	−2.44	−3.44	−2.09	

**Table 7:** Impact of reconstruction algorithms and modes on the image uniformity for scanning the Cathpan^®^ 504 phantom with the Halcyon accelerator equipped with CBCT and iCBCT. The results are shown for 0 and 5 min gaps between measurements (i_0 and i_5, respectively) for Pelvis, for 0 and ~6 min for Pelvis Large (i_0 and i_5, respectively) and for 1–5 series (s_1–s_5). VOI: 20 mm diameter, 5 mm height.

	Mean Value [HU]					Image Uniformity (±30 HU)					
Mode/Gap (i_0, i_5)/Series (s_1–s_5)	Center	Left	Top	Right	Bottom	Left (1)	Top (2)	Right (3)	Bottom (4)	Mean (1–4)	Mean (Series s_1–s_5
Cone-beam CT (CBCT)											
Pelvis i_0 s_1	7.52	1.29	1.16	−1.06	−1.73	−6.22	−6.35	−8.57	−9.25	−7.60	−7.41
Pelvis i_0 s_2	7.37	0.22	1.05	0.69	−1.33	−7.15	−6.32	−6.68	−8.70	−7.21	
Pelvis i_5 s_1	7.37	0.22	1.05	0.69	−1.33	−7.15	−6.32	−6.68	−8.70	−7.21	−8.04
Pelvis i_5 s_2	8.02	1.17	1.42	−1.23	−1.39	−6.85	−6.60	−9.26	−9.42	−8.03	
Pelvis i_5 s_3	8.74	−0.09	0.55	0.58	−1.25	−8.83	−8.19	−8.16	−9.99	−8.79	
Pelvis i_5 s_4	7.88	1.50	0.60	−1.48	−1.45	−6.38	−7.29	−9.36	−9.33	−8.09	
Pelvis i_5 s_5	7.95	0.05	1.00	0.27	−1.72	−7.90	−6.95	−7.69	−9.68	−8.06	
PelvisLarge i_0 s_1	7.99	4.37	5.56	4.54	2.51	−3.62	−2.42	−3.45	−5.47	−3.74	−4.17
PelvisLarge i_0 s_2	8.84	3.52	5.02	5.64	2.76	−5.32	−3.82	−3.20	−6.07	−4.60	
PelvisLarge i_5 s_1	10.20	2.51	2.73	0.46	−0.59	−7.69	−7.48	−9.75	−10.80	−8.93	−8.77
PelvisLarge i_5 s_2	9.86	1.29	2.44	1.65	−0.65	−8.58	−7.42	−8.21	−10.52	−8.68	
PelvisLarge i_5 s_3	9.79	2.44	2.46	0.36	−0.73	−7.35	−7.33	−9.43	−10.52	−8.66	
PelvisLarge i_5 s_4	9.92	1.33	2.31	1.75	−0.79	−8.59	−7.61	−8.17	−10.71	−8.77	
PelvisLarge i_5 s_5	9.74	2.27	2.15	0.20	−0.83	−7.48	−7.59	−9.55	−10.58	−8.80	
Iterative reconstruction CBCT (iCBCT)											
Pelvis i_0 s_1	2.76	−8.60	−9.12	−10.29	−10.05	−11.36	−11.87	−13.04	−12.81	−12.27	−12.43
Pelvis i_0 s_2	3.23	−8.32	−8.85	−10.04	−10.23	−11.55	−12.08	−13.27	−13.47	−12.59	
Pelvis i_5 s_1	4.25	−8.04	−7.77	−8.50	−9.48	−12.29	−12.03	−12.75	−13.73	−12.70	−12.42
Pelvis i_5 s_2	3.57	−7.29	−7.72	−9.59	−9.37	−10.86	−11.29	−13.16	−12.93	−12.06	
Pelvis i_5 s_3	3.62	−7.83	−7.43	−8.88	−9.46	−11.46	−11.05	−12.50	−13.08	−12.02	
Pelvis i_5 s_4	4.06	−7.52	−7.94	−9.20	−9.16	−11.57	−11.99	−13.26	−13.21	−12.51	
Pelvis i_5 s_5	4.02	−8.41	−7.91	−9.14	−9.64	−12.42	−11.93	−13.16	−13.66	−12.79	
PelvisLarge i_0 s_1	3.71	−3.45	−3.29	−3.90	−5.02	−7.16	−7.00	−7.61	−8.73	−7.62	−8.07
PelvisLarge i_0 s_2	4.69	−3.61	−3.27	−3.56	−4.88	−8.30	−7.96	−8.25	−9.57	−8.52	
PelvisLarge i_5 s_1	5.45	−4.03	−4.36	−5.48	−6.27	−9.49	−9.82	−10.94	−11.72	−10.49	−11.63
PelvisLarge i_5 s_2	5.87	−4.92	−4.74	−5.35	−6.80	−10.79	−10.61	−11.22	−12.66	−11.32	
PelvisLarge i_5 s_3	6.39	−4.76	−4.45	−6.03	−6.54	−11.14	−10.84	−12.41	−12.92	−11.83	
PelvisLarge i_5 s_4	6.23	−5.42	−4.99	−5.97	−7.10	−11.65	−11.22	−12.20	−13.33	−12.10	
PelvisLarge i_5 s_5	6.33	−4.94	−4.75	−6.91	−7.66	−11.27	−11.08	−13.24	−13.98	−12.39	

**Table 8:** Optimal procedures and parameters for determining the irradiation isocenter using PABIG^nx^ 3D dosimeter, Halcyon and TrueBeam accelerators and polyGeVero-CT data processing.

Accelerator	Reconstruction Algorithm	Mode	Signal Averaging	Scanning Time Interval [min]	Radiation Field Parameters	Fiducial Markers	Image Processing
TrueBeam	iCBCT	Pelvis	iCBCT: 1 CBCT: 5	0 (up to 5 scans)	2 mm MLC gap and 10,000 MU	shielded copper wire of the network cable	Image averaging (no or max from three series); Background subtraction; Filter: not obligatory
Halcyon		Pelvis/ Pelvis Large Fast		0 (up to 2 scans) 5 (up to 5 scans)			

## Data Availability

Data supporting the reported results are not stored in any publicly archived datasets. Readers may contact the corresponding author for further explanation of the results obtained.
